# Influence of sex and gender on the management of late-stage knee osteoarthritis

**DOI:** 10.1007/s12306-021-00725-8

**Published:** 2021-08-07

**Authors:** B. Moretti, A. Spinarelli, G. Varrassi, L. Massari, A. Gigante, G. Iolascon, M. G. Benedetti, A. M. Moretti

**Affiliations:** 1 Department of Medical Sciences of Basis, Neurosciences and Organs of Sense, General Hospital, Faculty of Medicine and Surgery, University of Study of Bari, Bari, Italy; 2 Department of Orthopedic and Trauma Unit, AOU Policlinico Consorziale Hospital, P.zza G. Cesare 11, 70124 Bari, BA Italy; 3 Paolo Procacci Foundation, Rome, Italy; 4 Department of Biomedical and Specialty Surgical Sciences, University of Ferrara, Ferrara, Italy; 5 Clinical Orthopedics, Department of Clinical and Molecular Science, Università Politecnica Delle Marche and Ospedali Riuniti Ancona, Ancona, Italy; 6 Department of Medical and Surgical Specialties and Dentistry, University of Campania “Luigi Vanvitelli”, Naples, Italy; 7 Physical Medicine and Rehabilitation Unit, IRCCS-Istituto Ortopedico Rizzoli, Bologna, Italy; 8 Italian Group for Health and Gender, Bari, Italy

**Keywords:** Gender, Osteoarthritis, Pain, Sex bias, Total knee arthroplasty

## Abstract

**Purpose:**

The exact nature of sex and gender differences in knee osteoarthritis (OA) among patient candidates for total knee arthroplasty (TKA) remains unclear and requires better elucidation to guide clinical practice. The purpose of this investigation was to survey physician practices and perceptions about the influence of sex and gender on knee OA presentation, care, and outcomes after TKA.

**Methods:**

The survey questions were elaborated by a multidisciplinary scientific board composed of 1 pain specialist, 4 orthopedic specialists, 2 physiatrists, and 1 expert in gender medicine. The survey included 5 demographic questions and 20 topic questions. Eligible physician respondents were those who treat patients during all phases of care (pain specialists, orthopedic specialists, and physiatrists). All survey responses were anonymized and handled via remote dispersed geographic participation.

**Results:**

Fifty-six physicians (71% male) accepted the invitation to complete the survey. In general, healthcare professionals expressed that women presented worse symptomology, higher pain intensity, and lower pain tolerance and necessitated a different pharmacological approach compared to men. Pain and orthopedic specialists were more likely to indicate sex and gender differences in knee OA than physiatrists. Physicians expressed that the absence of sex and gender-specific instruments and indications is an important limitation on available studies.

**Conclusions:**

Healthcare professionals perceive multiple sex and gender-related differences in patients with knee OA, especially in the pre- and perioperative phases of TKA. Sex and gender bias sensitivity training for physicians can potentially improve the objectivity of care for knee OA among TKA candidates.

## Background

Osteoarthritis (OA) is the leading cause of functional disability and reduced quality of life among adults in developed countries. OA of the knee accounts for more than 80% of the total disease burden [1] with an annual incidence of 181.2 per 100,000 population worldwide, representing a 8.2% increase from 1990 [2]. In a population-based study of older adults in Italy, 74% of individuals met the American College of Rheumatology clinical criteria for knee OA and 1/3 had some form of symptomatic peripheral OA [3].

The prevalence of knee OA continues to grow alongside increases in its risk factors such as age and obesity [4].

Nonetheless, the exact nature of sex and gender differences in knee OA and especially in-patient candidates for TKA remains unclear and requires better elucidation before it can influence clinical practice for the purpose of improving patient care.

Sex and gender interact in complex ways to affect health outcomes [5–7]; *sex* refers to biological differences between men and women that involves physical and physiological characteristics, including chromosomes, gene expression, hormone levels, and reproductive/sexual anatomy, that may play a role in the pathogenesis of disease and response to treatment. *Gender* refers to socially constructed characteristics such as norms, roles, and relations influenced by society and culture at the time. People develop their gender roles in response to the environment in which they grow up and live, in response to the upbringing they receive at home and at school, depending on family and peer group interactions and expectations and according to trends and fashions in the media. In addition, political affiliation, social status, and economic availability can modify gender roles, that in some societies are more rigid than those in others Gender contribute to differences in vulnerabilities and susceptibilities to illness, can influence experiences of crises and emergency situations, health behaviors and how illness is experienced; gender influences also access to healthcare, uptake of health services, and globally health outcomes [6].

Female sex is a well-established risk factor for knee OA in older adults [8–13]. Moreover, risk factors such as obesity are stronger predictors among women than men due to inflammatory and mechanical disease drivers [14]. Previous studies indicate that women over the age of 50 have a higher prevalence of knee OA and experience greater functional disability compared to men of the same age, but the reasons for this difference are poorly understood [15, 16]. Painful knee OA is also an independent predictor of mortality in middle-aged women [17].

Among available treatments, total knee arthroplasty (TKA) is a safe and cost-effective option that alleviates both pain and functional limitation related to end-stage knee OA [18–20]. Emerging data suggest that sex and gender-related differences in knee OA may also exist surrounding treatment. Despite a predilection for female sex, fewer women with symptomatic OA report discussing possible treatment with a physician than men, and women undergoing TKA tend to be treated at a later stage of disease associated with higher severity and functional disability than men [21, 22]. A more recent investigation reported that women have significantly higher healthcare utilization related to knee OA in the 12 months preceding TKA than men [23]. In terms of symptoms, a body of literature has established important sex and gender differences in the experience of pain, both related to physiological or biochemical differences [24] and psychological coping strategies [25], as well as sex differences in responses to pharmacological treatment [26]. With regard to TKA outcomes, one study reported that female sex was an independent risk factor for nonhome discharge but a protective factor against post-procedural complications [27].

Here, we performed a survey of healthcare professionals in Italy who attend candidates for TKA during all phases of care (pain specialists, orthopedic specialists, and physiatrists) in order to inform physician perceptions about the influence of sex and gender on knee OA presentation and management.

## Methods

### Study design

This study was a survey of physicians who treat patient candidates for TKA during all phases of care (preoperative, perioperative, and postoperative/rehabilitative) in Italy. Survey questions were designed to focus on the influence of sex and gender on knee OA presentation, care, and outcomes of TKA. Questions were elaborated by a multidisciplinary scientific board composed of 1 pain specialist, 4 orthopedic specialists, 2 physiatrists, and 1 expert in gender medicine. The survey was active for a period of 8 weeks (July 6, 2020–September 1, 2020) and communicated to a panel of physicians via e-mail using a password-protected web link. All survey responses were anonymized and handled via remote dispersed geographic participation.

### Survey

The survey included 5 demographic questions and 21 survey questions about common practices and perceptions related to the influence of sex or gender on symptomology and pain severity, pharmacological therapy, postoperative outcomes, rehabilitation strategy, and patient satisfaction (see “Appendix” for list of survey questions). Question responses were multiple-choice and either single response or multiple response depending on the nature of the question. Responses were tabulated descriptively (number and percentage) for the overall group and by physician specialty. For questions that allowed open-ended response justifications, comment responses were analyzed thematically.

## Results

### Respondent characteristics

The survey was sent to 122 healthcare professionals in Italy; of these, 56 (46%) accepted the invitation to participate and six respondents provided incomplete survey responses. Professional categories included anesthesiologists and pain specialists (9 respondents), physiatrists (17 respondents), orthopedic specialists (23 respondents), and the scientific board (7 respondents). Most respondents were male (71%), aged between 41–60 years (53%), worked in university settings (57%) and were geographically located in the south of Italy (46%). Twenty-seven percent of respondents saw up to 10 patients with knee OA or knee replacement per month, 37% saw between 11 and 20 patients, and 36% saw more than 20 patients per month.

### Preoperative symptomatology and pain severity

Sixty-seven percent of healthcare professionals disagreed with the assertion that male patients exhibit a more severe symptomatology than women upon first presentation (Q1), whereas 29% agreed with this assertion, and 4% were in total agreement. In contrast, 74% of respondents agreed or totally agreed that, among candidates with equivalent clinical and functional characteristics, women presented with poorer subjective impressions of functional limitation than men (Q2).

**Fig. 1:**
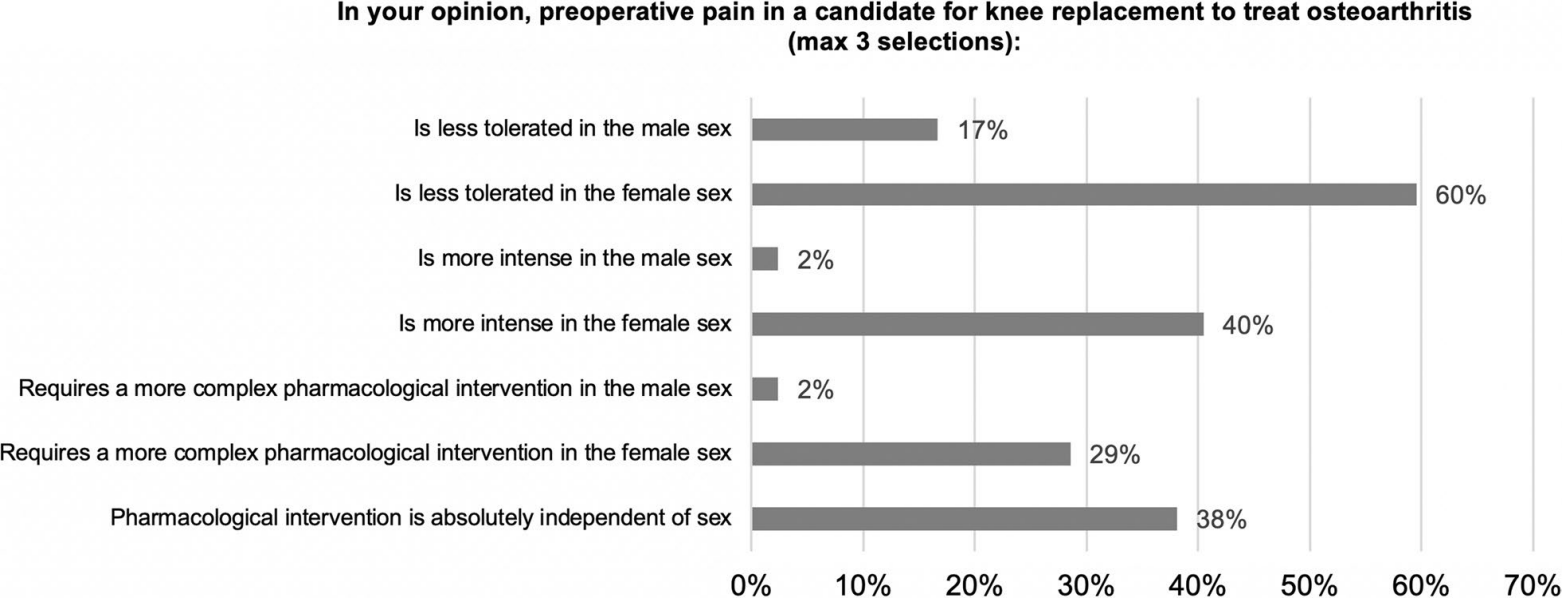
Sex differences in preoperative pain severity among candidates for knee replacement. Responses to survey question 3. Respondents could provide a maximum of 3 selections

When characterizing preoperative pain severity among candidates for knee replacement (Q3), the overall impression was that pain is less tolerated by female patients (60%), whereas 38% expressed that pain is more intense in female patients, and an equivalent proportion expressed that pain intensity is absolutely independent of sex (Fig. 1). Forty percent of respondents indicated that there was no bearing of sex on subsequent pharmacological intervention, while 29% suggested that more complicated pharmacological interventions are required in women. When responses to this answer were divided by physician specialty, pain specialists unanimously expressed that pain is less tolerated, more intense, and requires more complex pharmacological intervention in women. Conversely, physiatrists were in lesser agreement about a sex difference in the experience and subsequent treatment of knee OA pain.

Eighty percent of respondents agreed that socioeconomic and cultural characteristics influence the perception of pain (Q4).

### Preoperative pharmacological therapy

Eighty-six percent of respondents affirmed that the presence of comorbidities influenced their therapeutic and/or rehabilitative choices in patients with knee OA, independent of sex (Q5). Only 40% agreed that there are sex or gender differences in responses to available pain treatments in candidates for TKA (Q6). In the sub-analysis by physician specialty, 74% of physiatrists disagreed with this statement. When respondents were asked to qualify their “yes” responses to this question, most specified that they were referring to non-steroidal anti-inflammatory drugs (NSAIDs), opioids, and generally lower efficacy of analgesics among women compared to men. Conversely, Ninety% of respondents disagreed that the prescription of pharmacological treatment is guided by gender-specific indications (Q7).

**Fig. 2:**
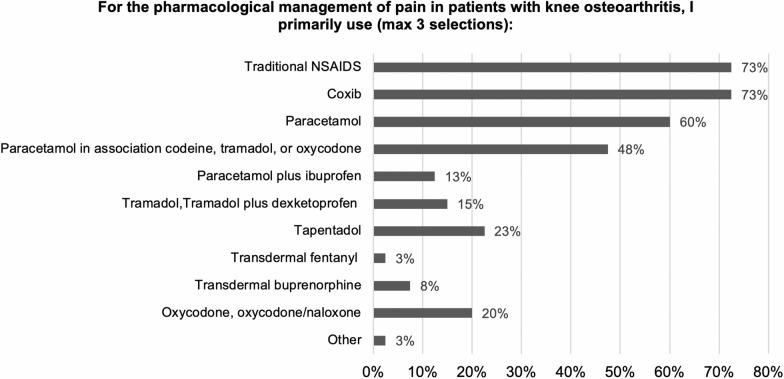
Pharmacological management of pain in knee OA. Responses to survey question 8. Respondents could provide a maximum of 3 selections

With regard to the pharmacological agents prescribed by respondents to treat pain related to knee arthritis, NSAIDs and Coxib were most commonly used by 73% of respondents, followed by paracetamol (60%) and opioid-paracetamol combinations (48%; Q8, Fig. 2). Noteworthy, pain therapists were more likely to use tramadol alone or in combination (50%), whereas orthopedic specialists and physiatrists primarily used NSAIDs, Coxib, and paracetamol. The most commonly prescribed oral analgesics (excluding opioids) were Coxib (83%), diclofenac (65%), and paracetamol (55%; Q9). For opioids, 41% only prescribed opioids in patients with knee OA when pain was refractory to other treatments and 18% reserved opioids for moderate to severe knee pain (Q10). When responses were analyzed by physician specialty, pain therapists tended to use opioids for moderate to severe knee pain whereas physiatrists and to some extent orthopedic specialists reserved opioids for cases of refractory pain.

Finally, 45% of respondents agreed or totally agreed that in the preoperative phase, sex and gender-related differences influence responses to pharmacological treatment for pain management among patients with knee OA (Q11). Only pain specialists reflected higher agreement with the presence of a sex or gender difference, whereas physiatrists disagreed.

### Postoperative outcomes

**Fig. 3:**
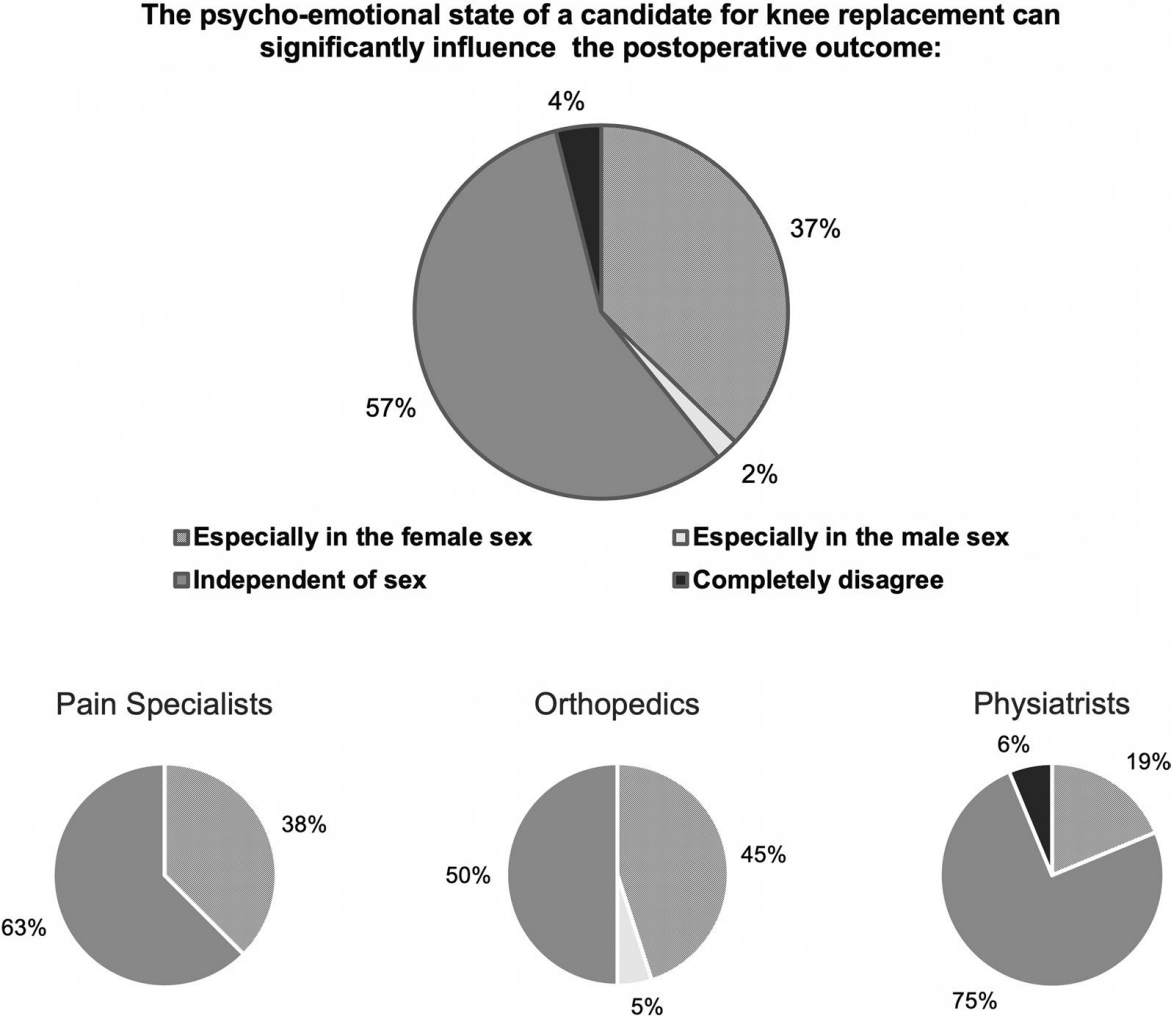
Psycho-emotional state and postoperative outcomes after TKA. Responses to survey question 15 for the overall group and by physician specialty

Eighty-five percent of respondents agreed that early and correct management of pain and inflammation in the perioperative phase can positively influence the final outcome of TKA independent of sex (Q12), while 13% expressed that this was especially true among female patients. In contrast, 61% agreed that, among patients with similar clinical, functional, radiographic, and psycho-emotive characteristics, gender can significantly influence clinical outcomes of knee replacement (Q13). When responses were divided by physician specialty, a gender difference was most heavily supported by pain specialists (88%) and to some degree orthopedics (65%). Forty-seven percent of respondents agreed that chronic knee pain in the preoperative period (> 18 months) predicts pain around the prosthesis independent of sex; 35% disagreed with the statement and 18% agreed especially in female patients (Q14). Finally, 57% agreed that the psycho-emotional state of TKA candidates can significantly influence postoperative outcomes independent of sex (Q15). Thirty-seven percent asserted that this was especially true in female patients. When responses were divided by physician specialty, pain therapists, and orthopedic specialists were most likely to support a sex difference (Fig. 3).

### Rehabilitation strategy

Sixty-two percent of respondents agreed that there were possible sex differences in the clinical and functional results of postoperative rehabilitation after knee replacement (Q16). Yet, 76% disagreed that postsurgical treatment and rehabilitation should be guided by gender-specific indications (Q17). Respondents who agreed with this statement specified compliance as an important gender-specific difference, as well as level of pain and muscle condition or bone health prior to surgery. In a question administered only to physiatrists, 73% disagreed that gender-specific “analgesic” physiotherapy techniques are useful for postoperative rehabilitation (Q18).

### Postoperative patient satisfaction

Fifty percent of respondents agreed that dissatisfaction after total knee replacement occurs independent of sex, whereas the other half qualified that dissatisfaction is more frequent in female patients (Q19). When divided by physician specialty, pain therapists, and orthopedic specialists were more likely to indicate a female predilection whereas physiatrists overwhelmingly asserted that treatment dissatisfaction is independent of sex (87%).

Finally, 74% of respondents agreed that current literature regarding TKA is potentially biased by the use of scales that are not validated for gender-specific indications (Q20).

## Discussion

The present survey of healthcare professionals indicated that physicians who treat candidates for TKA, especially pain specialists and orthopedic specialists, perceive significant sex and gender differences in disease presentation, symptom management, and treatment responses. Although these perspectives are partly supported by available literature, the survey responses emphasized a need to focus more clearly on what sex and gender differences truly mean for the clinical management of knee OA. The absence of measures and treatments that take into consideration gender-specific indications could represent a missed opportunity for improved patient care.

The interaction of pain and gender/sex is an emerging and extremely complex research topic [28–30]. In general, the drivers of sex differences in musculoskeletal pain can be divided into 3 categories: (1) differences in pain sensitivity and reporting, (2) differences in socioeconomic and psychological factors, and (3) sex-linked predisposing biological factors. First, literature data indicate that men and women have different responses to pain, where women are more variable in their responses, have increased pain sensitivity and have a predisposition to a larger number of painful disorders relative to men [31]. Women are also more likely to report pain than men and are, therefore, more frequently perceived as “emotional” or “overreactive”, a stereotype that leads physicians to mistrust or disregard complaints of pain [32]. The interaction of higher pain sensitivity and reporting in women and physician mistrust is an important factor that may in part underlie observations that women often arrive later than men to surgery for knee OA. It is also noteworthy that women report higher postoperative pain [33], which in itself may be related to an interplay of higher pain sensitivity and late presentation for surgery with higher disease severity and disability [21, 22]. Second, and also pertinent to the later observation, women are subject to different social pressures and accordingly develop divergent psychological coping mechanisms compared to men. In some cultural contexts, many women retain traditional roles in the household (e.g., cleaning, cooking, caring for sick family members) and may delay seeking care or interventions that interfere with these roles for a prolonged period of time. Women are also less likely to have a caregiver at home and instead are often the primary caregivers for family members with disability [34]. With regard to psychological coping strategies for pain, a study of 72 men and 96 women with knee OA found that higher pain and physical disability in women were mediated by catastrophizing score (using the Catastrophizing Scale of the Coping Strategies Questionnaire) [25]. Another large study found that maladaptive responses including catastrophizing were associated with worse preoperative pain and function measures in candidates for TKA [35]. Thirdly, sex-linked predisposing factors for knee OA in women include stronger predictive value of risk factors such as obesity [14], differences in cartilage composition and walking mechanics compared to men [36, 37]. differences in knee kinematics during gait both before and after TKA compared to men [38, 39], a higher annual rate of tibial and patellar cartilage loss than men [40, 41], and the presence of estrogen receptors in human articular cartilage [42]. The loss of estrogen during menopause is associated with a decrease in life-long painful conditions such as headache and migraine, but can be associated with additional painful conditions such as osteoporosis and joint inflammation [43]. In summary, it is likely that perceptions of lower pain tolerance and higher pain intensity among women reported by healthcare professionals in our survey reflect a plethora of factors contributing to a true clinical difference between men and women that merits further definition in a context of knee OA.

Another important opinion expressed by physicians and especially pain specialists in our survey was that women may require a different (for example, more personalized) pharmacological approach and/or experience less efficacy of pharmacological pain treatment or poorer outcomes of TKA compared to men. Sex differences in analgesic responses have been reported wherein female sex and gender tend to be associated with lesser analgesia [33, 44]. Women are also prescribed and consume more medications than men [31]. Considering this evidence, a potentially useful approach in a context of knee OA is multimodal analgesia involving classes of analgesics that interact additively or synergistically [45–47]. This approach is historically associated with shorter hospitalization time, better recovery, and reduced healthcare costs [48]. The use of weak opioids with other agents such as NSAIDs, Coxib, or paracetamol can have an opioid-sparing effect and limit adverse events that may complicate the management of knee OA [49]. In contrast, the influence of gender or sex on TKA outcomes is less clear: although female sex was an independent risk factor for nonhome discharge in one study [27], another study found no differences in the outcomes of TKA between men and women in an Asian population [50]. A lack of definitive evidence for a sex or gender difference in TKA outcomes may be related to socioeconomic and cultural factors as well as the interaction of multiple factors with sex and gender as described above.

Another intriguing idea emerging from the survey was the importance of assessing pain and prescribing treatment using gender and sex-specific indications. In response to Q26, a majority of respondents agreed that the absence of measures validated for gender-specific indications is a shortcoming of available studies on knee OA. At present, there are no specific validated tools available to facilitate an approach tailored by sex/gender in knee OA or chronic pain in general. The development of such tools may aid future investigations of the influence of sex and gender on pain as well as the detection of sex differences in a clinically meaningful manner that can inform physician decision-making.

Having defined inherent sex and gender differences contributing to divergent disease presentation in OA, it is equally important to examine biases among healthcare professionals and sex differences in these perceptions. First, it is noteworthy that a majority of respondents to our survey were male. Inherent sex or gender bias among male physicians has been systematically investigated: in interview studies, male physicians perceived men seeking medical help as brave and stoic, whereas women were described as sensitive, complaining, or hysterical [32]. To this extent, a previous study founded that prescriber characteristics significantly influenced pain treatment in men and women [51]. Male physicians in particular may benefit from gender bias sensitivity training, and the effects of such training should be investigated in future research efforts to improve the objectivity of healthcare surrounding knee OA. Second, our survey findings revealed distinct impressions about sex and gender differences in knee OA across physician specialties. Physiatrists were less likely to affirm sex and gender differences in a context of knee OA than pain specialists or orthopedic specialists. This may be in part related to the fact that pain specialists and orthopedic specialists were more likely to be male in our survey and partly related to the phase in which physicians encounter patients with knee OA. Pain specialists and orthopedic specialists are likely to see patients at peak disease and pain severity, when well-established sex differences in OA presentation and pain are more evident (e.g., a tendency of women present at a later stage of disease with higher peak pain that is more refractory to pharmacological treatment). Physiatrists who treat patients in the rehabilitative phase are probably witnessing a different kind of pain (i.e., pain related to physical rehabilitation) that may not be subject to the same influences of sex or gender or may be similarly intense between the two sexes. It would be useful to clarify possible sex or gender differences in pain during the rehabilitative phase after knee replacement.

The present study had a few limitations. First, the nature of this study as a survey best informs physician perceptions and not necessarily the actual nature of sex differences in knee OA, although many trends observed in our study are supported by available literature. Second, as aforementioned, a majority of respondents were male, consistent with the demographic characteristics of the included physician specialties (especially orthopedic specialists, who are more likely to be male) in Italy. Future investigations may consider balancing the sex of survey participants to better inform perceived sex and gender differences in healthcare settings. Third, this survey was restricted to physicians in Italy and may reflect some cultural bias. A recent decree (law 3/2018 article 3) calls for the integration of gender medicine into the practices of the Italian National Health Service, but a lack of education and training among physicians nationwide represents an important obstacle to its successful implementation. Finally, it should be noted that, while sex and gender differences in knee OA have important bearing on its clinical management, they represent only one dimension of a multifactorial influence on outcome comprised of socioeconomic status, comorbidities, and other variables. Studies should consider whether disparities are due to sex or gender differences in disease incidence, disease expression or severity, or additional factors by examining a variety of inequity types. Further research is needed to better inform the influences of these factors individually and together on the management and outcomes of knee OA.

## Conclusions

In conclusion, the results of our survey demonstrate that healthcare professionals perceive multiple sex and gender-related differences in patients with knee OA, especially in the pre and perioperative phases of TKA. The exact nature and significance of these differences between men and women should be better investigated with regard to differences in disease severity; clinical factors at initial presentation; physical, biochemical, and psycho-emotive characteristics; and socioeconomic as well as cultural factors. At the same time, it is necessary to evaluate bias in physician perceptions about sex and gender. In Italy [52]and worldwide, the study of gender differences and the development of gender-based medicine are considered relevant to the advancement of life sciences. Sex and gender analysis can help reveal and assess inequality and can improve actions to create institutional change to ensure greater equity Future efforts are necessary to synthesize these observations into clinically translatable sex- or gender-specific indications to improve care in patients with knee OA.

## Data Availability

The datasets used and/or analyzed during the current study are available from the corresponding author on reasonable request.

## Appendix (Questions)

1. Express your agreement or disagreement with the following statement: “At the first specialist visit for knee arthritis, male patients present with more severe symptomology than female patients.”
  - Completely disagree
  - Disagree
  - Agree
  - Totally agree
2. Express your agreement or disagreement with the following statement: “In patients with equivalent clinical and functional characteristics, female patients with knee arthritis present with a poorer subjective perception of functional limitation than male patients.”
  - Completely disagree
  - Disagree
  - Agree
  - Totally agree
3. In your opinion, preoperative pain in a candidate for knee replacement to treat osteoarthritis: (indicate a maximum of 3 options below)
  - The intensity is absolutely independent of sex
  - Is more intense in the female sex
  - Is more intense in the male sex
  - Is less tolerated in the female sex
  - Is less tolerated in the male sex
  - Pharmacological intervention is absolutely independent of sex
  - Requires a more complex pharmacological intervention in the female sex
  - Requires a more complex pharmacological intervention in the male sex
4. Express your agreement or disagreement with the following statement: “The socioeconomic and cultural characteristics of a patient influence their perception of pain.”
  - Completely disagree
  - Disagree
  - Agree
  - Totally agree
5. Does the presence of comorbidities in patients with osteoarthritis of the knee influence your choice of therapy and/or rehabilitation?
  - No
  - Yes, especially for the female sex
  - Yes, independent of sex
  - Yes, especially for the male sex
6. Do sex or gender differences influence the response to available pain treatments in candidates for knee replacement? (If yes, specify.)
  - Yes
  - No
7. Is the prescription of pharmacological treatment guided by gender-specific indications? (If yes, specify.)
  - Yes
  - No
8. For the pharmacological management of pain in patients with knee osteoarthritis, I primarily use: (indicate a maximum of 3 options below)
  - Oxycodone, oxycodone/naloxone
  - Transdermal buprenorphine
  - Transdermal fentanyl
  - Tapentadol
  - Tramodol plus dexketoprofen
  - Tramadol
  - Paracetamol plus ibuprofen
  - Paracetamol in association with opioids (codeine, tramadol, or oxycodone)
  - Paracetamol
  - Coxib
  - Traditional NSAIDs
  - Other
9. Regarding oral analgesics (excluding opioids), patients with knee osteoarthritis most frequently use: (indicate a maximum of three options below)
  - Diclofenac
  - Paracetamol plus NSAIDs
  - Paracetamol
  - Ibuprofen
  - Coxib
  - Naproxen
  - Other
10. I prescribe opioids for pain management in patients with knee osteoarthritis:
  - I never prescribe opioids in these patients
  - I only prescribe weak opioids as needed
  - In patients with knee osteoarthritis and moderate to severe pain
  - Only during the preoperative period to favor early rehabilitation
  - Only for short periods in all patients
  - Only in the presence of pain refractory to other treatments
  - Only during the perioperative phase (24–72 h)
  - Other
11. Express your agreement or disagreement with the following statement: “In the preoperative phase, sex or gender differences influence the response to pharmacological treatment for pain management in patients with knee osteoarthritis.”
  - Completely disagree
  - Disagree
  - Agree
  - Totally agree
12. The early and correct perioperative management of pain and inflammation can positively influence the final outcome:
  - Especially in female patients
  - Especially in male patients
  - Independent of sex
13. Express your agreement or disagreement with the following statement: “In patients with equivalent clinical/functional scores, psycho-emotive status, and radiographic characteristics, gender differences can significantly influence postoperative clinical outcomes.”
  - Completely disagree
  - Disagree
  - Agree
  - Totally agree
14. Patients with a history of chronic knee pain preoperatively (> 18 months) are predisposed to pain around the prosthesis after knee replacement.
  - Especially in female patients
  - Especially in male patients
  - Independent of sex
  - I completely disagree with the statement
15. The psycho-emotional state of a candidate for knee replacement can significantly influence the postoperative outcome.
  - Especially in female patients
  - Especially in male patients
  - Independent of sex
  - I completely disagree with the statement
16. Express your agreement or disagreement with the following statement: “The clinical and functional results of postoperative rehabilitation after knee replacement can vary by sex.”*
  - Completely disagree
  - Disagree
  - Agree
  - Totally agree
  *Question not administered to pain therapists
17. In patients with knee replacement, do you believe that the choice of postsurgical rehabilitative treatment should be guided by gender-specific indications? (If yes, specify.)
  - Yes
  - No
18. Do you find gender-specific analgesic physiotherapy techniques to be useful during the postoperative rehabilitation phase? (If yes, specify.)*
  - Yes
  - No
  *Question not administered to pain therapists or orthopedics.
19. Dissatisfaction after complete knee replacement:
  - Is more frequent in male patients
  - Is more frequent in female patients
  - Is independent of sex
20. Express your agreement or disagreement with the following statement: “The various positive and negative results of knee replacement published in the literature could be biased by the use of scales that are not validated for gender-specific indications.
  - Completely disagree
  - Disagree
  - Agree
  - Totally agree
  - I don’t know
